# Restriction of Rift Valley Fever Virus Virulence in Mosquito Cells

**DOI:** 10.3390/v2020655

**Published:** 2010-02-17

**Authors:** Valerie M. Vaughn, Cale C. Streeter, David J. Miller, Sonja R. Gerrard

**Affiliations:** 1 Medical Scientist Training Program, School of Medicine, University of Michigan, Ann Arbor, Michigan, USA; E-Mail: valmv@umich.edu (V.M.V.); 2 Department of Epidemiology, School of Public Health, University of Michigan, Ann Arbor, Michigan, USA; E-Mail: calesz@umich.edu (C.C.S.); 3 Department of Microbiology and Immunology, School of Medicine, University of Michigan, Ann Arbor, Michigan, USA; E-Mail: milldavi@med.umich.edu (D.J.M.); 4 Internal Medicine School of Medicine, University of Michigan, Ann Arbor, Michigan, USA

**Keywords:** arbovirus, arthropod, NSs, non-structural protein, innate immunity, cytopathic effect, virulence factor

## Abstract

Arboviruses are maintained in a natural cycle that requires blood-sucking arthropod and vertebrate hosts. Arboviruses are believed to persistently infect their arthropod host without overt pathology and cause acute infection with viremia in their vertebrate host. We have focused on elucidating how a specific arbovirus, Rift Valley fever (RVF) virus, causes cytopathic effect in cells derived from vertebrates and non-cytopathic infection in cells derived from arthropods. We demonstrate that the vertebrate virulence factor, NSs, is functional in arthropod cells but is expressed at significantly lower levels in infected arthropod *versus* infected vertebrate cells.

## 1. Introduction

Rift Valley fever (RVF) virus is a mosquito-borne virus of the *Bunyaviridae* family, *Phlebovirus* genus, endemic to sub-Saharan Africa [1]. RVF virus causes disease in humans, as well as domestic ruminants such as cattle and sheep [1,2]. In humans, RVF is typically a self-limited febrile illness, although severe disease such as hemorrhagic fever and encephalitis, also occurs in a small percentage of human cases [1,2]. RVF in domestic ruminants results in abortion and high rates of mortality, especially in very young animals [1,2]. Localized flooding creates habitat for floodwater mosquitoes and is the initiating factor in RVF epizootics [3,4]. As a result, RVF epizootics are predictable weeks in advance based on satellite weather data [5,6]. The fact that RVF activity is predictable suggests that vaccination campaigns could be targeted to areas with imminent risk thereby allowing for prevention of epizootics.

*Culex* and *Aedes* species mosquitoes are thought to be the most important vectors for transmission of RVF virus during epizootics [3,7,8]. It is not known where RVF virus resides during inter-epizootic periods, however transovarial transmission has been demonstrated in field-caught *A. mc intoshi* (reported originally as *A. lineatopennis)* [3,9]. Despite the critical role mosquitoes have in transmission, and presumably in maintenance, of RVF virus, very little is known about the replication strategy of this virus in mosquitoes. Much of the molecular details of RVF virus replication and virus-host interactions were obtained from studies performed in either vertebrate cell culture or vertebrate animals. In vertebrates, RVF virus infection is acute and lytic [2]. By contrast, RVF virus is thought to cause a non-lytic persistent infection of mosquitoes [3]. While it is believed that most arboviruses cause little or no detrimental effect on their natural mosquito host [10], RVF virus has been shown to decrease egg-laying, re-feeding efficiency and the lifespan of *C. pipiens* [11,12], a mosquito species that is known to vector RVF virus in the wild.

The genome of RVF virus comprises three single-stranded RNA segments [13]. The S segment is ambi-sense and encodes for a non-structural protein (NSs) in the viral genomic sense (vRNA) and the nucleocapsid protein (N) in the viral genomic copy sense (cRNA) [13]. NSs is an indirect and a direct inhibitor of type I interferon (IFN) signaling in vertebrate cells. NSs down-regulates vertebrate host cell mRNA synthesis by sequestering components of a basal transcription factor complex, TFIIH [14]. As a consequence, β-IFN and type I IFN-regulated genes are not expressed in response to virus infection [15]. NSs directly blocks IFN signaling through interaction with SAP30, which represses transcription of β↕IFN [16]. NSs has also been recently shown to prevent RNA-activated protein kinase (PKR) from down-regulating translation in the presence of dsRNA [17,18]. While IFN signaling pathways are not present in mosquitoes, TFIIH is present, therefore it is possible that NSs acts as a transcriptional inhibitor in mosquitoes.

We report on a comparison of RVF virus production and the synthesis of RVF virus proteins in arthropod and vertebrate cells. The envelope glycoproteins and N accumulate similarly regardless of source animal. However, NSs is expressed at significantly lower levels in arthropod cells as compared to vertebrate cells. The low level of NSs expression provides a mechanism for how RVF virus-infected mosquitoes escape down-regulation of basal transcription and suggests an explanation for the extreme diversity observed amongst the NSs of phleboviruses.

## 2. Results

### 2.1. RVF Virus Can Productively Infect Vertebrate and Arthropod Cells

**Figure 1.**
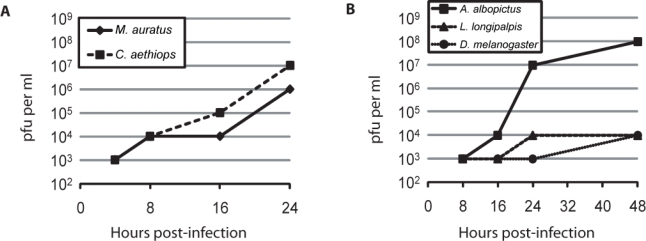
**RVF virus productively infects vertebrate and arthropod cells. A.** *M. auratus* and *C. aethiops* and, **B.** *A. albopictus*, *L. longipalpis* and *D. melanogaster* cells were infected with RVF virus at an MOI of 1. The vertebrate lines were grown at 35 °C and the arthropod lines at 28 °C. Media was collected from the cells at the indicated times and the virus titer in the media was determined by plaque assay on *C. aethiops* cells.

Hamster (*Mesocricetus auratus*) [19], monkey (*Cercopithecus aethiops*) [20], mosquito (*A. albopictus*) [21], sandfly (*Lutzomyia longipalpis*) [22] and fruitfly (*Drosophila melanogaster*) [23] cells were infected with RVF virus at an MOI of 1. The vertebrate and arthropod cell lines were grown at 35 °C and 28 °C, respectively. Supernatants were collected at 4, 8, 16 and 24 hpi for vertebrate and 8, 16, 24, and 48 hpi for arthropod cell lines. The collected virus was titered on *C. aethiops* cells. The cells were not washed following infection, therefore the initial timepoint in both the vertebrate and arthropod cells reflects the residual inoculum (Figure 1A and 1B). In both vertebrate cell lines, virus production was first observed at the 8 h timepoint, and continued out to the final timepoint at 24 h (Figure 1A). Extensive cytopathic effect (CPE) was observed at 24 h in both vertebrate cell lines, therefore no further timepoints were taken (data not shown). The arthropod cell lines required more time to secrete virus than vertebrate cells, with virus release first observed at 16 h in *A. albopictus* cells (Figure 1B). Amongst the arthropod lines, *A. albopictus* cells secreted the highest final titers with the most rapid kinetics (Figure 1B). This result was expected since RVF virus has been shown to productively infect *A. albopictus* [24,25]. *L. longipalpis* cells took 24 h to produce virus and only increased the titer by 10^1^ pfu/mL over baseline (Figure 1B). Although RVF virus can infect *L. longipalpis* following intra-thoracic inoculation, this sandfly species was only marginally competent for transmission [26]. The *D. melanogaster* cells yielded similar results to the *L. longipalpis* cells and virus production was not evident until 48 hpi (Figure 1B). No CPE was observed with any of the arthropod lines (data not shown).

### 2.2. Kinetics of RVF Virus Structural Protein Expression

Production of the structural proteins N and Gn was monitored by immunofluorescence microscopy. Images were obtained for fields selected at random and total number of cells, infected cells, and infected cells that express Gn were counted as discussed in the materials and methods section. Evidence of infection was observed in greater than 85% of cells by 8 hpi in both vertebrate cell lines. Approximately 90% of the infected vertebrate cells expressed Gn (Table 1). By 24 hpi, nearly 100% of the vertebrate cells were infected and expressed Gn. By contrast, *A. albopictus* cells displayed a high percentage of infected cells (>95%) throughout the timecourse, however the percentage of infected cells expressing Gn was lower (Table 1). Only 51% of infected cells expressed Gn at 8 h, and expression did not peak until 24 h (Table 1). The slower rate of Gn expression in *A. albopictus* cells appears to correlate with slower virus production relative to the vertebrate cell lines (Figure 1B). All of the arthropod cell lines showed an approximately 10% reduction in Gn expression between 24 h and 48h. Others have reported that the switch between acute and persistent phases of bunyavirus infection in insect cells occurs around 24 h [27–30], therefore this reduction in Gn positive infected cells could be because virion production is beginning to be down-regulated.

### 2.3. NSs Is Expressed at Very Low Levels in Arthropod Cell Lines

**Figure 2.**
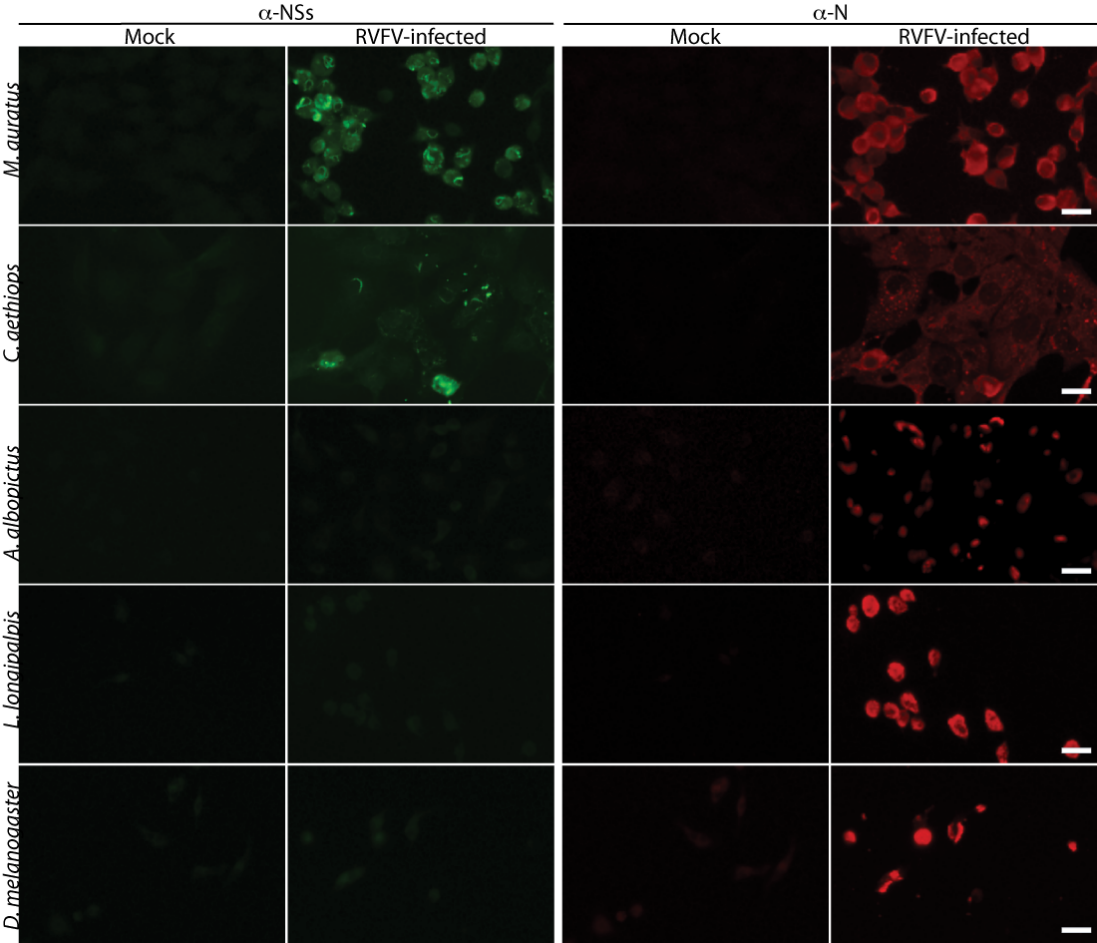
**RVF virus infected arthropod cells express less NSs relative to vertebrate cells.** *M. auratus* and *C. aethiops*, *A. albopictus* and *L. longipalpis* cells were infected with RVF virus at an MOI of 1. The vertebrate lines were grown at 35 °C and the arthropod lines at 28 °C. The images shown are from the 24 h and 48 h timepoints for the vertebrate and arthropod cells, respectively. The NSs and N antibody complexes were labeled with anti-mouse 488 (green) and anti-rabbit 594 (red), respectively. Scale bars represent 20 μm.

Production of the vertebrate virulence factor, NSs, was monitored by immunofluorescence microscopy. Cells were labeled with rabbit anti-N and mouse anti-NSs antibodies. Figure 2 represents images obtained from randomly selected fields of cells for the vertebrate and arthropod cell lines. In agreement with previous studies, filamentous structures that labeled with NSs antibody were localized to the nucleus in both vertebrate cell lines (Figure 2) [14,31,32]. At 8 hpi, greater than 65% of infected *C. aethiops* and *M. auratus* cells expressed NSs (Table 2). Expression of NSs increased to nearly 90% of infected cells by 24 h in both *C. aethiops* and *M. auratus* cells (Table 2). By contrast, the vast majority of RVF virus-infected arthropod cells displayed NSs staining that was only slightly above the level found in mock-infected cells (Figure 2 and Table 2). Interestingly, although filamentous NSs staining was occasionally observed, the filaments were never found to be associated with nuclei (Table 2 and data not shown).

The low level expression of NSs in the vast majority (≥98%) of arthropod cells (Table 2) made it difficult to identify positive cells upon visual inspection. Therefore, we analyzed the average intensity of NSs expression per cell as described in the materials and methods section. RVF virus-infected *A. albopictus* cells had an average cell intensity 2.1-fold mock, whereas infected *M. auratus* cells had an average cell intensity 7.3-fold mock. These data demonstrate that there is a clear difference in NSs expression level between arthropod and vertebrate cells.

### 2.4. NSs is Not Detected in Mosquito Cells by Immunoprecipitation

**Figure 3.**
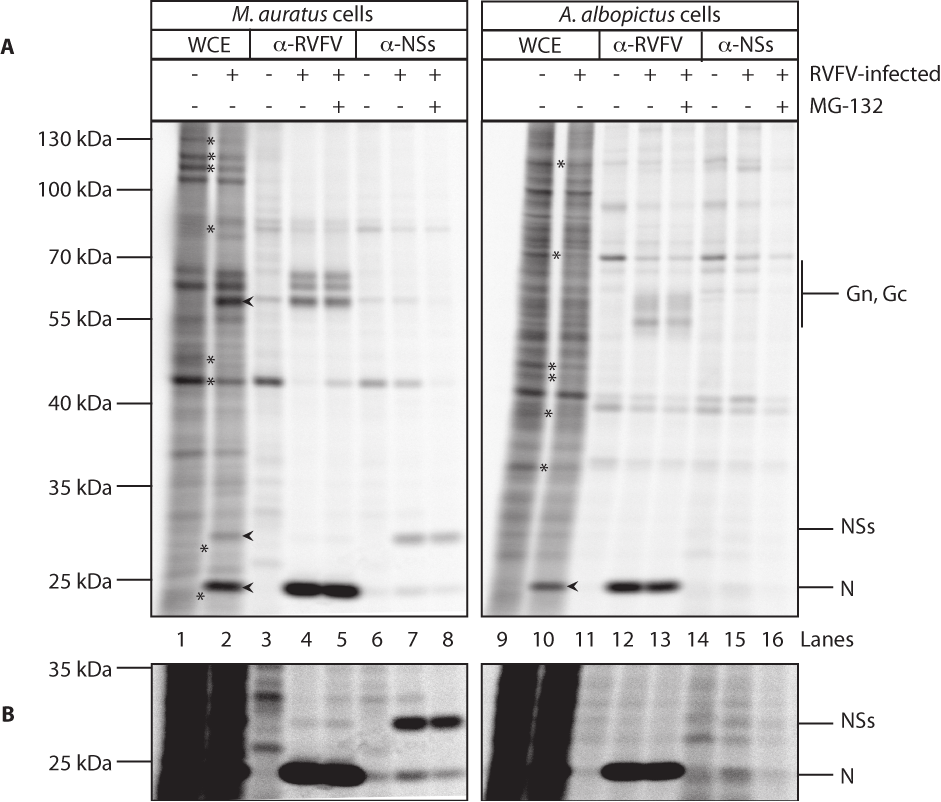
**RVF virus infected** ***A. albopictus*** **cells synthesize lower amounts of NSs protein relative to** ***M. auratus*** **cells.** *M. auratus* and *A. albopictus* cells were infected with RVF virus at an MOI of 1. At 20 hpi, proteins were labeled for 1 h with [^35^S]-methionine and cysteine either in the presence or absence of the proteasome inhibitor MG-132. Viral proteins were immunoprecipitated with either an anti-RVF virus or anti-NSs antibody. An aliquot of labeled whole cell extract (WCE) was also run on the same 10% SDS-PAGE gel. **A.** The numbers on the left of the figure refer to the positions of molecular weight markers. On the right of the gel the positions of the envelope glycoproteins Gn and Gc, N and NSs are indicated. The asterisks indicate protein bands that are more intense in the WCE from mock-infected cells than RVF virus-infected cells. The arrowheads indicate the virus proteins in the WCE from infected cells. **B.** A darker image of the lower portion of the gel shown in A.

The low levels of NSs expression in arthropod cell lines could be due to low-levels of synthesis or rapid turnover of the protein. In order to distinguish between these possibilities, protein synthesis was assayed in *M. auratus* and *A. albopictus* cells by radioactive labeling of newly synthesized proteins. Additionally, a proteasome inhibitor (MG-132) was used in order to determine if low levels of NSs in arthropod cells were the result of degradation in the proteasome. Cells were either mock-infected or infected with RVF virus at an MOI of 1. At 20 hpi, proteins were labeled for 60 min with ^35^S cysteine and methionine in either the presence or absence of MG-132. Labeled proteins were immunoprecipitated with either mouse anti-RVF virus (Figure 3A, lanes 3–5 and 11–13) or mouse anti-NSs (Figure 3A, lanes 6–8 and 14–16) followed by separation by SDS-PAGE.

Additionally, an aliquot of crude whole cell extract (WCE) from mock and RVF virus infected cells was run on the same gel (Figure 3A, lanes 1–2 and 9–10). A darker image of the lower portion of the gel is shown in Figure 3B. In *M. auratus* cells, N and the envelope glycoproteins were immunoprecipitated by the polyclonal RVF virus antibody (Figure 3A, lanes 4–5) and these same bands were also prominent in the WCE lane (Figure 3A, lane 2 arrowheads), indicating that the cells are infected and actively synthesizing viral proteins. The mouse anti-RVF virus antibody does not appear to recognize NSs. When the mouse anti-NSs antibody is used, a band of approximately 30 kDa is apparent in infected cells (Figure 3A, lanes 7–8) but not mock infected (Figure 3A, lane 6) and this same band can also be observed in the infected WCE lane (Figure 3A lane 2 arrowhead). In agreement with previously published results [33], no differences were observed in the amounts of protein immunoprecipitated from cells that were labeled in the presence or absence of MG-132 (Figure 3A, compare lanes 4 and 5 and lanes 7 and 8) indicating that the proteasome does not contribute to the degradation of viral structural proteins. The band intensities for N and NSs were quantified, as described in the materials and methods section, and the ratio of immunoprecipitated N to NSs for *M. auratus* cells was 27:1. This value is not a result of differences in relative cysteine and methionine content as N and NSs have 1 and 5 cysteine residues, respectively, and both proteins have 12 methionines. While this ratio is in part a function of the efficiency of immunoprecipitation with the two antibodies, it is obvious from the infected cell extract that more N is made relative to NSs. Therefore, although N and NSs genes are on the same genomic segment (S), the proteins are synthesized at dramatically different levels in *M. auratus* cells.

*A. albopictus* cells were productively infected as indicated by the presence of N in the WCE (Figure 3A, lane 10 arrowhead) and N and envelope glycoproteins in the polyclonal RVF virus antibody lanes (Figure 3A, lanes 12–13). The envelope glycoproteins, Gn and Gc, migrate faster when the virus is grown in *A. albopictus* cells rather than *M. auratus* cells (Figure 3A, compare lanes 4 and 12). This likely reflects structural differences in N- linked glycosyl groups between vertebrates and arthropods [34,35]. In contrast to the results obtained with *M. auratus* cells, no NSs was detected in the anti-NSs immunoprecipitate lanes (Figure 3A and 3B, lanes 15–16) or infected WCE lane (Figure 3A, lane 10). Although the level of N in *A. albopictus* cells is lower than that obtained for *M. auratus* cells, NSs would have been detectable if it were made at the same level relative to N (27:1) as that found for *M. auratus* cells. Given the detection limit associated with this experiment, levels of NSs relative to N in *A. albopictus* cells are at minimum 2.2-fold lower than those found in *M. auratus* cells. Similar to the results found in *M. auratus* cells, MG-132 did not significantly affect the amounts of viral proteins in *A. albopictus* cells (Figure 3A, compare lanes 12 and 13 and lanes 15 and 16), indicating that the proteasome does not play a major role in viral protein degradation.

The NSs of ZH548-MP12, the same strain of RVF virus as used in this study, is capable of shutting down global host protein synthesis in *C. aethiops* cells and appears to be the sole viral protein required for this effect [36]. Based on radiolabel incorporation in the WCE (Figure 3A, lanes 1–2 and 9–10) we observed a ∼40% and ∼25% reduction in protein synthesis in RVF virus-infected cells *M. auratus* and *A. albopictus* cells, respectively. Additionally, several proteins appeared to be visibly down-regulated in infected *M. auratus* and *A. albopictus* cells (asterisks in Figure 3A). While we do not know why cellular protein synthesis is reduced in RVF virus-infected *A. albopictus* cells, our immunofluorescence results (Figure 2 and Table 2) indicate that a small amount of NSs is made in *A. albopictus* cells. Additionally, others have successfully detected NSs from ZH548-MP12-infected mosquito cells by immunoprecipitation utilizing different antibodies, longer labeling times and a different mosquito cell line (AP-61 cells, derived from *A. pseudoscutellaris*) [37].

### 2.5. NSs Expression is Not Temperature-Sensitive

**Figure 4.**
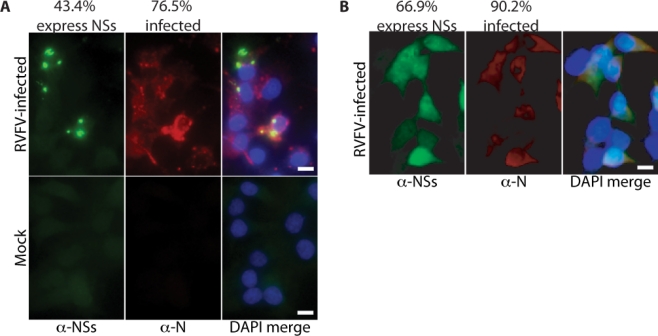
**NSs expression is not temperature-sensitive.** *M. auratus* cells were infected with RVF virus at an MOI of 1. The cells were grown at **A.** 28 °C for 24 h or **B.** 35 °C for 8 h. The NSs and N antibody complexes were labeled with anti-mouse 488 (green) and anti-rabbit 594 (red), respectively and the nuclei with DAPI (blue). Scale bars represent 10 μm.

The differences observed with respect to NSs expression could be due to the fact that the arthropod cell lines were grown at 28 °C and the vertebrate cell lines were grown at 35 °C. Thus, we tested the possibility that NSs expression is temperature-sensitive. *M. auratus* cells were infected with RVF virus at an MOI of 1 at 28 °C. At 24 hpi, cells were fixed and stained with rabbit anti-N and mouse anti-NSs antibodies. The percentage of infected cells that expressed NSs was ∼43% (Figure 4A). Although the percentage of infected cells that expressed NSs was lower than that obtained when *M. auratus* cells were grown at 35 °C, it still represents a much higher percentage than observed in arthropod cells. Notably, the NSs did not localize to the nucleus and rather was found in puncta within the cytoplasm (Figure 4A). NSs is normally found in the cytoplasm early in an infection (≤8 hpi) (Figure 4B). Thus, the pattern of NSs staining for cells grown at 28 °C is potentially due to a cold-sensitive step in NSs trafficking to the nucleus. Therefore, in *M. auratus* cells NSs nuclear localization is temperature-sensitive, but NSs expression is not.

### 2.6. Plasmid-Expressed NSs in Mosquito and Fruitfly Cells Accumulates in the Nucleus

**Figure 5.**
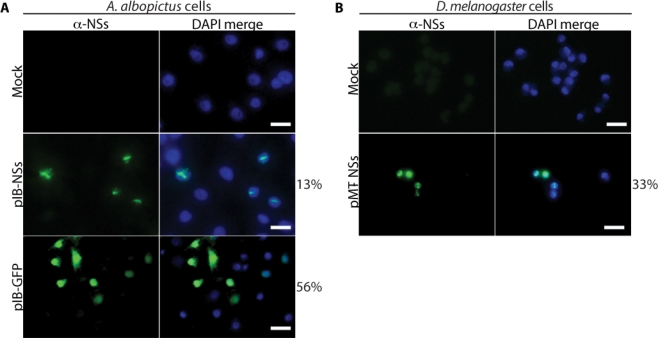
**Arthropod cells transfected with a NSs expression plasmid efficiently express NSs. A**. *A. albopictus* cells were transfected with an empty vector (Mock), NSs expression plasmid (pIB-NSs), or GFP expression plasmid (pIB-GFP). Cells were grown at 28 °C for 36 h. **B.** A stable *D. melanogaster* cell line was developed using an NSs expression plasmid with a copper-inducible metallothionein promoter (pMT-NSs). Expression was induced with 1 mM CuSO4 and cells were then grown at 28 °C for 16 h. The NSs antibody complex was labeled with anti-mouse 488 (green) and the nuclei with DAPI (blue). Scale bars represent 20 μm.

*A. albopictus* cells were transfected with empty vector, NSs expression plasmid or GFP expression plasmid. At 36 h post-transfection, cells receiving either mock or NSs expression plasmid were fixed and stained with mouse anti-NSs. Cells transfected with the GFP expression plasmid were used to control for transfection efficiency. NSs expression was observed in ∼13% of cells that were transfected with the NSs expression plasmid and the protein localized to nuclear filaments (Figure 5A). A stable *D. melanogaster* cell line with a NSs expression plasmid was also analyzed. The *D. melanogaster* cells showed expression of NSs as nuclear filaments in 33% of cells (Figure 5B). Therefore, both *A. albopictus* and *D. melanogaster* cells are competent to express NSs and the expressed protein localizes to the nucleus. Similar results have been obtained for expression of NSs using a Semliki Forest virus-derived expression system in AP-61 cells (derived from *A. pseudoscutellaris*) [38].

### 2.7. Plasmid-Expressed NSs Inhibits Reporter Gene Expression in Fruitfly Cells

**Figure 6.**
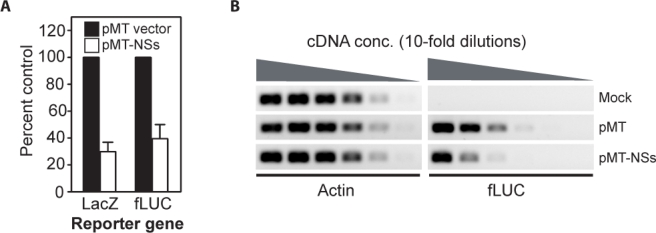
**Reporter gene expression is inhibited by NSs in** ***D. melanogaster*** **cells. A**. *D. melanogaster* cells were transfected with expression plasmids for either luciferase (pS2MT-LUC) or β-galactosidase (pS2MT-LacZ). In addition to the reporter plasmid, cells received either empty plasmid (pMT) or NSs expression plasmid (pMT-NSs). Expression of all genes was controlled by a copper-sensitive promoter (metallothionein). Reporter levels were measured at 24 h post-induction. The error bars reflect the standard errors of the mean for three independent experiments. **B**. *D. melanogaster* cells were transfected with pMT (Mock) or pS2MT-LUC and either empty plasmid (pMT) or NSs expression plasmid (pMT-NSs). RNA was harvested and both actin and luciferase mRNA levels were examined by semi-quantitative RT-PCR.

To assess whether RVF virus NSs was functional in insect cells, *D. melanogaster* cells were transfected either with a luciferase or a β-galactosidase expression plasmid (pS2MT-LUC or pS2MT-LacZ), and either empty expression plasmid (pMT) or NSs expression plasmid (pMT-NSs). Expression from the metallothionein promoter (MT) was induced with copper sulfate and reporter levels were measured at 24 h post-induction (Figure 6A). Expression of NSs resulted in a 60–70% reduction in reporter expression relative to the empty expression plasmid control (Figure 6A). Levels of luciferase mRNA were also measured by semi-quantitative RT-PCR (Figure 6B). The luciferase mRNA was approximately 10-fold lower in NSs expressing cells, relative to the vector alone control. These data suggest that RVF virus NSs is capable of acting as an inhibitor of transcription in *D. melanogaster*cells.

### 2.8. Discussion

The NSs of RVF virus is expressed at a much lower level in arthropod cells than in vertebrate cells. Most significant is the low level of expression found in mosquito cells that were derived from *A. albopictus* [21], a mosquito that is competent for RVF virus transmission [24,25]. The reduced level of NSs expression cannot be explained by temperature differences between growth optima of vertebrate and arthropod cells nor can it be explained by inherent instability of NSs in arthropod cells. The most likely explanations are that the mRNA for NSs is either produced at lower levels than in vertebrate cells or that it is unstable. It is also possible that the NSs mRNA is not efficiently translated.

Comparison of all of the phlebovirus proteins within strains of a particular virus and across virus species indicates that NSs exhibits the highest degree of variation [39,40]. In fact, the average percentage of amino acid identity for pairwise NSs comparisons across the phleboviruses transmitted by mosquitoes and sandflies is ∼19% [40]. By comparison, N, which is encoded on the same genomic segment, yields average pairwise comparison values of ∼54% [40]. Clearly, NSs does not experience the same selective pressures as N. A strain of RVF virus, known as Clone 13, has a large internal deletion within the NSs ORF which removes ∼90% of the coding sequence [39,41]. This strain is a plaque-purified clone derived from a strain (74HB59) isolated from a non-fatal human case of RVF [41]. Clone 13 is avirulent in rodents and does not productively infect vertebrate cells that are competent for IFN signaling [33,41,42]. However, Clone 13 is capable of growing in *A. albopictus* cells (C6/36) [24,43] and can produce a disseminated infection in several *Culex* and *Aedes* species following either an infectious bloodmeal or intra-thoracic inoculation [24,41,43]. Therefore, NSs is not required for growth of RVF virus in mosquitoes and our data indicate that NSs is poorly expressed in *A. albopictus* cells. It is possible that NSs plays little or no role in maintenance of RVF virus in the mosquito host, and thus the main selective pressure on the NSs gene in the mosquito host is for reduced expression.

Interestingly, it has been demonstrated that expression of dsRNA derived from either the complete S segment or the N gene severely reduces the ability of RVF virus to replicate in cells derived from *A. pseudoscutellaris* [38]. By contrast, expression of dsRNA derived from the NSs gene had no effect on the ability of RVF virus to replicate in these cells [38]. These results were interpreted as a failure of NSs RNA to induce an effective RNAi response against the virus [38]. However, another interpretation of these data is that an effective RNAi response was mounted, however degradation of NSs mRNA does not adversely affect virus replication in *A. pseudoscutellaris* cells. Along these lines, there is no data from viruses of the *Bunyaviridae* family that addresses whether the encapsidated genome and antigenome are sensitive to degradation mediated by interfering RNAs [38,44,45]. All published data on RNAi-mediated restriction of bunyavirus replication can be interpreted as being primarily caused by degradation of mRNAs essential for production of the replicative proteins (RNA-dependent RNA polymerase and/or N) [38,44,45]. We hypothesize that genome and anti-genome are not sensitive, since base-pairing of the interfering RNAs with the genome would be hindered by bound N. Our interpretation is supported by the fact that Clone 13 can replicate in both mosquitoes and cells derived from mosquitoes [24,41,43]. Assessing the levels of NSs mRNA in the presence or absence of NSs dsRNA would indicate which interpretation is correct.

In order to maintain a persistent infection in arthropod hosts, there must be strong selective pressure on arboviruses to minimize the detrimental effects of replication and accessory proteins, such as NSs. Bunyamwera virus (family *Bunyaviridae*, genus *Orthobunyavirus*) also has an NSs that is involved in down-regulation of vertebrate host cell transcription [46]. The Bunyamwera virus NSs is encoded in an ORF that overlaps that of the N gene, and shares no sequence homology with RVF virus NSs [13]. This protein is expressed in arthropod cells, however in contrast to its effect on vertebrate cells, it does not down-regulate transcription [46,47]. Our data suggests that a different mechanism exists for overcoming the detrimental effects of RVF virus NSs on arthropod cells. RVF virus NSs is poorly expressed in arthropod cells, but the protein is functional, as indicated by reduced reporter gene expression in *D. melanogaster* cells and by the decrease in protein synthesis in *A. albopictus* cells. RVF virus NSs has been shown to interact with human p44 and XPD, both components of the basal transcription factor, TFIIH [14]. NSs sequesters human p44 and XPD in filaments within the nucleus and as a result other TFIIH proteins fail to localize to the nucleus and are degraded in the cytoplasm [14]. Expression of NSs from a plasmid in vertebrate [14,32] and arthropod cells (Figure 5) [37,38] results in nuclear localization of NSs, and in some cells, formation of filaments. NSs does not appear to have a nuclear localization signal (NLS), and it is believed to enter the nucleus by virtue of the NLS on p44 [14,32]. It is not yet known whether the NSs of RVF virus binds arthropod p44. However, *C. quinquefasciatus*, *A. aegypti* and *D. melanogaster* p44 are 50–53% identical and 67–69% similar to human p44. Many arboviruses encode proteins that are involved in down-regulation of host gene expression (reviewed in [48]). Several of these proteins have demonstrated activity in cells derived from their arthropod host, although presumably their main purpose is to counteract mammalian innate immune responses (reviewed in [48]). Since most arboviruses persistently infect their arthropod hosts, there will no doubt be many other mechanisms uncovered in the future for the blunting of the detrimental effects of these host gene expression inhibitors in arthropods.

Arboviruses must replicate efficiently in disparate hosts in order to be maintained in nature. Therefore, it is not surprising that many have been shown to adapt to growth in one of their hosts through acquisition of specific amino acid changes [49]. Our results indicate that RVF virus NSs is differentially expressed in vertebrate and arthropod cells and this differential is likely critical for its fitness in each host. To our knowledge, the differential expression of NSs represents the first example of phylum-specific expression of an arbovirus protein. The mechanism by which NSs is expressed at lower levels in arthropod cells as compared to vertebrate cells is not yet known. However our results are consistent with either a failure to accumulate NSs mRNA or a failure to translate NSs mRNA. NSs can form nuclear filaments when expressed from a plasmid in *A. albopictus* and *D. melanogaster* cells (Figure 5), or from a Sindbis virus-based expression system in *A. pseudoscutellaris* cells [38], demonstrating that the poor expression and failure to form nuclear filaments in RVF virus-infected arthropod cells is either a property of the virus or due to an activity in arthropod cells that only manifests in the context of infection.

NSs appears to contribute to the RVF virus–induced cytopathology in vertebrate cells [36]. A recombinant RVF virus strain that lacked NSs was found to produce just as much virus as the parental strain but with less cytopathology in Vero E6 (*C. aethiops*) cells [36]. RVF virus has been shown to cause morbidity and mortality in *C. pipiens* [11,12,50]. We observed an overall decrease in protein synthesis and identified proteins that were obviously down-regulated in infected *A. albopictus* cells (Figure 3A). Additionally, we have found that over a 72 h timecourse, *A. albopictus* cells infected with RVF virus remain viable but fail to proliferate (CCS and SRG, unpublished data). It is possible that RVF virus persistence in mosquitoes is dependent on low levels of NSs expression to lessen the detrimental effects of transcriptional inhibition. The sole function of the RVF virus NSs gene may be to counteract the innate immune response of mammals in order to generate the prolonged viremia necessary for transmission to naïve mosquitoes [10]. If this is the case, then strains of RVF virus that lack the ability to express NSs should be less detrimental to mosquitoes than strains that can express NSs. Furthermore, strains that over-express NSs would be expected to result in increased mortality of mosquitoes. These predictions have important implications for the development of live-attenuated vaccines against RVF virus. NSs inhibits pathways in vertebrates that have no correlates in arthropods, notably the ability to inhibit PKR and β↕IFN signaling. Thus, it may be possible to make a vaccine strain of RVF virus that is attenuated in mammalian hosts but is virulent for mosquitoes.

## 3. Experimental Section

### 3.1. Cells and Virus

The *M. auratus* cells (BSR-T7/5) were obtained from Dr. K. Conzelmann (Max-von Pettenkofer-Institut, Munchen, Germany), the *C. aethiops* cells (Vero E6) from the Centers for Disease Control and Prevention (Atlanta, GA), the *A. albopictus* (C6/36) and *D. melanogaster* (S2) cells were from the American Type Culture Collection (Manassas, Virginia), and the sandfly cells (LL-5) from Dr. I. Novella (University of Toledo). The ZH-548 MP-12 vaccine strain of RVF virus was obtained from Dr. R. Tesh (World Reference Center of Emerging Viruses and Arboviruses) and was handled under BSL-2 conditions.

### 3.2. Antibodies

The hybridomas for the NSs (3C3-1-1) and Gn (R1-4D4-1-1) antibodies were obtained from Dr. G. Ludwig at the United States Army Medical Research Institute of Infectious Diseases (USAMRIID). The mouse anti-RVF virus antibody was obtained from Dr. P. Rollin (CDC). The rabbit anti-N antibody was developed against bacterially expressed full-length N.

### 3.3. Construction of pIB-GFP, pIB-NSs and pMT-NSs

The GFP gene was amplified with Taq polymerase using primers GFPNSSDel5 (5′-GATATCAATGGTGAGCAAGGGCGAGGAG-3′) and GFPNSSdel3 (5′-GATATCTTACTTGTACAGCTCGTCCAT-3′), then cloned into pIB/V5-His TOPO® TA. The template for amplification of the NSs gene was pTrRVFV-S, which represents a full-length clone of the S segment of the ZH-501 strain of RVF virus [51]. In order to make pIB-NSs, the NSs gene was amplified with Taq polymerase using primers NSSBAM5 (5′-GGATCCATGGATTACTTTCCTGTG-3′) and NSSXHO3 (5′-CTCGAGCTAATC AACCTCAACAAATCC-3′), then cloned into pIB/V5-His TOPO® TA. In order to make pMT-NSs, the NSs gene was amplified with Taq polymerase using primers RVFNSsSpe5 (5′- ACTAGTATGGATTACTTTCCTGTG -3′) and pMTRVFnssXho3 (5′- CTCGAGCTAATCAACCTCAACAAATC-3′), and cloned into pCR-TOPO®, then subcloned into the pMT-V5/HisA.

### 3.4. Plasmids and Transfection

The pVAX1, pIB/V5-His TOPO® TA, pCR-TOPO® and pMT-V5/HisA plasmids were obtained from Invitrogen Corp. The construction of the NSs and GFP expression plasmids, pIB-NSs, pIB-GFP and pMT-NSs, is described in supplemental methods. The reporter plasmids, pS2MT-LUC and pS2MT-LacZ, have been described elsewhere [52]. *A. albopictus* cells were plated at a density of 1.0 x10^5^ cells per well on glass coverslips in 24-well culture plates. Cells were transfected using *Trans*IT®-LT1 (Mirus Bio, LLC, Madison, WI) 24 h after plating. Plasmids were used at 2.5 μg per well and transfectant at 2.5 μL per well. *D. melanogaster* cells were transfected and expression induced as described previously [52].

### 3.5. Infections and Virus Titration

Confluent cell monolayers of *M. auratus*, *C. aethiops*, *A. albopictus*, or *L. longipalpis* cells on glass coverslips in 24-well plates were infected with RVF virus at an MOI of 1. Vertebrate cells were incubated at 35 °C and arthropod cells at 28 °C, unless otherwise indicated. Media was harvested at the timepoints indicated in the text and stored at −80 °C. Cells were fixed and processed for immunofluorescence as described elsewhere [53]. In order to determine virus concentration, harvested media was thawed and serially diluted. Dilutions were used to infect confluent *C. aethiops* monolayers grown in 96-well plates. Plates were scored for cytopathic effect (CPE) at 1 week post-infection.

### 3.6. Quantification of Indirect Immunofluorescence Microscopy Data

Images were obtained utilizing an Olympus BX fluorescence microscope equipped with a digital camera. For each cell line and timepoint, two randomly selected fields were photographed. Cells were counted using the Cell Counter Plug-in for ImageJ (NIH) using the following methodology to determine percent expression of N, Gn and NSs. DAPI positive cells were marked and counted to determine the total cell count. The DAPI markers were overlaid on the corresponding N image and positive cells were marked and counted, thus providing the percentage of total cells that express N. For all experiments the percentage of cells expressing N was arbitrarily equated with percentage of cells infected with RVF virus. The N markers were overlaid on the corresponding NSs image. N positive cells that were also NSs positive were marked and counted, thus providing the percentage of infected cells that express NSs. The methodology was repeated to obtain the percentage of infected cells that express Gn.

### 3.7. Intensity Quantification of NSs Expression

Using Adobe Photoshop, the green channel from the NSs image was merged with the corresponding red channel from the N image. Cell outlines for individual cells and small groups of cells were selected on the red channel (N). The corresponding selection was cut from the green channel (NSs) and pasted into a new image with a black background. A histogram was created for the intensity of green fluorescence (NSs) using the Color Histogram utility in ImageJ. The total intensity of green fluorescence (NSs) from the histogram was divided by the number of cells selected to determine an average intensity of green fluorescence (NSs) per cell, therefore providing a relative value of NSs expression.

### 3.8. Radioactive Immune-Precipitation Assay

Confluent monolayers of *M. auratus* and *A. albopictus* cells in 6-well plates were infected with RVF virus at an MOI of 1. Proteins were labeled at 16 hours post-infection (hpi), and immunoprecipitated as previously described [54]. Immune precipitated proteins were resolved by SDS-PAGE, the gel was fixed, dried and placed in a PhosphoImager cassette. Screens were scanned with a Storm PhosphoImager and band intensities were quantified using ImageQuant software (Amersham Pharmacia Biotech, Piscataway N.J.).

### 3.9. Reporter Gene Expression

Enzymatic assays to determine β-galactosidase and luciferase levels were done as previously described [52]. For RT-PCR, total RNA was isolated with Trizol, digested with RQ1 DNAse, repurified with RNAsy columns per the manufacturer's instruction, and quantiated by spectrophotometry and gel electrophoresis. For reverse transcription, Superscript II (Invitrogen) was used in concert with 2.5 μg total RNA and oligo-dT primers per the manufacturer's instructions. PCR reactions were done with 10-fold serial cDNA dilutions, 200 nM gene-specific primers, and 30 cycles.

## 4. Conclusions

The RVF virus virulence factor, NSs, is differentially expressed in cells derived from arthropods *versus* vertebrates. The envelope glycoproteins and N accumulate similarly regardless of source animal. The low level of NSs expression provides a mechanism for how RVF virus-infected mosquitoes escape down-regulation of basal transcription and suggests an explanation for the extreme diversity observed amongst the NSs of phleboviruses. Reduced NSs expression may correlate with persistence, and thus these results have implications for development of live-attenuated vaccines against RVF.

## Figures and Tables

**Table 1.** Gn expression in RVF virus infected cells.

**HPI**	**Source Specie (Cell Line)**	**Total Cells**	% Infected^*^	**% Infected expressing Gn**
8	*M. auratus* (BSR-T7/5)	393	85.0%	89.8%
16		790	99.1%	99.5%
24		365	99.5%	100%
48^†^				
8	*C. aethiops* (Vero E6)	609	83.1%	92.7%
16		344	96.5%	100%
24		295	97.3%	100%
48^†^				
8	*A. albopictus* (C6/36)	529	97.9%	51.4%
16		655	98.0%	63.1%
24		380	96.3%	87.7%
48		547	96.3%	76.5%
8	*L. longipalpis* (LL-5)	n.d.	n.d.	n.d.
16		69	100%	92.0%
24		114	99.1%	99.1%
48		193	97.9%	85.7%
8	*D. melanogaster* (S2)	n.d.	n.d.	n.d.
16		229	93.0%	91.5%
24		272	98.5%	96.3%
48		97	92.8%	85%

Notes:

(*) N expression was equated with RVF virus infection.

(†) Cytopathic effect prevented counting of 48 h timepoint. n.d. Not determined.

**Table 2.** NSs expression in RVF virus infected cells.

**HPI**	**Source Specie (Cell Line)**	**Total Cells**	**% Infected**	**% Infected expressing NSs**
8	*M. auratus* (BSR-T7/5)	398	90.2%	66.9%
16		577	100%	88.4%
24		855	100%	92.7%
48^†^				
8	*C. aethiops* (Vero E6)	324	90.1%	69.9%
16		309	98.7%	86.2%
24		418	98.1%	89.8%
48^†^				
8	*A. albopictus* (C6/36)	378	97.4%	0.272%^‡^
16		482	96.1%	0.648%^‡^
24		393	95.4%	1.07%^‡^
48		328	91.2%	0.669%^‡^
8	*L. longipalpis* (LL-5)	138	71.7%	2.02%^‡^
16		104	98.1%	1.96%^‡^
24		227	94.7%	0.465%^‡^
48		189	95.2%	1.11%^‡^
8	*D. melanogaster* (S2)	n.d.	n.d.	n.d.
16		288	95.1%	0.00%^‡^
24		279	92.8%	0.00%^‡^
48		83	100%	0.00%^‡^

Notes:

(*) N expression was equated with RVF virus infection.

(†) Cytopathic effect prevented counting of 48 h timepoint.

(‡) Due to the low expression levels only cells exhibiting NSs filaments were counted. n.d. Not determined.
